# Early Celastrol Administration Prevents Ketamine-Induced Psychotic-Like Behavioral Dysfunctions, Oxidative Stress and IL-10 Reduction in The Cerebellum of Adult Mice

**DOI:** 10.3390/molecules24213993

**Published:** 2019-11-05

**Authors:** Stefania Schiavone, Paolo Tucci, Luigia Trabace, Maria Grazia Morgese

**Affiliations:** Department of Clinical and Experimental Medicine, University of Foggia, Viale Pinto, 1 71122 Foggia, Italy; stefania.schiavone@unifg.it (S.S.); paolo.tucci@unifg.it (P.T.); mariagrazia.morgese@unifg.it (M.G.M.)

**Keywords:** ketamine, psychosis, cerebellum, celastrol, oxidative stress, NADPH oxidases

## Abstract

Administration of subanesthetic doses of ketamine during brain maturation represents a tool to mimic an early insult to the central nervous system (CNS). The cerebellum is a key player in psychosis pathogenesis, to which oxidative stress also contributes. Here, we investigated the impact of early celastrol administration on behavioral dysfunctions in adult mice that had received ketamine (30 mg/kg i.p.) at postnatal days (PNDs) 7, 9, and 11. Cerebellar levels of 8-hydroxydeoxyguanosine (8-OHdG), NADPH oxidase (NOX) 1 and NOX2, as well as of the calcium-binding protein parvalbumin (PV), were also assessed. Furthermore, celastrol effects on ketamine-induced alterations of proinflammatory (TNF-α, IL-6 and IL-1β) and anti-inflammatory (IL-10) cytokines in this brain region were evaluated. Early celastrol administration prevented ketamine-induced discrimination index decrease at adulthood. The same was found for locomotor activity elevations and increased close following and allogrooming, whereas no beneficial effects on sniffing impairment were detected. Ketamine increased 8-OHdG in the cerebellum of adult mice, which was also prevented by early celastrol injection. Cerebellar NOX1 levels were enhanced at adulthood following postnatal ketamine exposure. Celastrol *per se* induced NOX1 decrease in the cerebellum. This effect was more significant in animals that were early administered with ketamine. NOX2 levels did not change. Ketamine administration did not affect PV amount in the cerebellum. TNF-α levels were enhanced in ketamine-treated animals; however, this was not prevented by early celastrol administration. While no changes were observed for IL-6 and IL-1β levels, ketamine determined a reduction of cerebellar IL-10 expression, which was prevented by early celastrol treatment. Our results suggest that NOX inhibition during brain maturation prevents the development of psychotic-like behavioral dysfunctions, as well as the increased cerebellar oxidative stress and the reduction of IL-10 in the same brain region following ketamine exposure in postnatal life. This opens novel neuroprotective opportunities against early detrimental insults occurring during brain development.

## 1. Introduction

The recreational use of the N-methyl-D-aspartate receptor (NMDA-R) antagonist ketamine, at subanesthetic doses, has been widely reported to cause psychedelic effects in humans [1]. Moreover, the development of a psychotic-like state has also been described following prolonged assumption of this psychoactive compound [2,3]. Despite an increasing scientific interest in ketamine’s psychotogenic effects, the mechanisms underlying the pathological contribution of this NMDA-R antagonist in psychosis development need to be further elucidated. In this context, the administration of subanesthetic doses of ketamine to rodents represents a reliable tool to mimic neuropathological alterations reminiscent of those observed in psychotic patients, in terms of biomolecular alterations, neurochemical dysfunctions and behavioral impairment [4]. Indeed, in rodents, increased locomotor activity and decreased discrimination abilities have been respectively associated with the agitation and disorganized behavior, as well as with the cognitive impairment observed in subjects suffering from psychosis [5,6,7]. Moreover, abnormalities in social behavior, such as withdrawal and decreased interactions, have been related to negative symptoms observed in psychotic patients [8]. 

Numerous lines of evidence have considered the psychotic disease to be the final result of a series of events occurring during the early stages of brain development [9]. Hence, animal models obtained by administering ketamine during a crucial period of central nervous system (CNS) maturation, such as the second postnatal week of life [10], might provide information on the possible pathogenetic contribution of an early insult to an enduring psychotic state in adulthood. 

Together with the widely known role of the prefrontal cortex in the pathogenesis of psychosis, in recent years, an emerging interest has been directed towards a possible implication of cerebellum in the development of this mental disorder [11,12]. Indeed, preclinical, clinical, neuroanatomical and neuroimaging reports began to highlight its important role not only in motor function regulation but also in the modulation of emotional and cognitive processes [13,14,15,16]. Structural cerebellar abnormalities, such as deficits in its gray matter volume, have also been described in antipsychotic-naive schizophrenic patients [17]. Moreover, vascular insults occurring in this brain region resulted in the onset of unremitting psychosis [18]. 

Administration of subanesthetic doses of ketamine in both early life stages and adult life has been widely reported to reduce the amount of the calcium-binding protein parvalbumin (PV) in different brain regions, such as prefrontal cortex and hippocampus [19,20,21,22]. However, poor evidence is available on the effects of early ketamine administration on cerebellar amount of PV, which has been shown to play a key role in regulating several physiological processes in this brain region [23], such as cell firing, synaptic transmission, as well as the resistance to neuronal degeneration following a variety of acute or chronic insults [24,25].

Oxidative stress, defined as an imbalance between reactive oxygen species (ROS) production and the antioxidant defenses of the cells, has been described as a key player in the pathogenesis of several CNS diseases, going from neurodegenerative to neuropsychiatric disorders [26], including psychosis [27]. The family of the Nicotinamide Adenine Dinucleotide Phosphate (NADPH) oxidase (NOX) enzymes represents one of the major ROS sources in the CNS, where it is involved in several physiological functions [28]. In particular, enhanced levels of NOX1 enzyme have been reported in neuropsychiatric diseases characterized by psychotic symptoms [29,30], and increased NOX2 expression was observed in specific brain regions, such as the prefrontal cortex and nucleus accumbens of environmental [19,31,32] and pharmacologic rodent models of psychosis, including the one obtained by ketamine administration in adult mice [33,34,35]. NOX1 and NOX2 mRNA and proteins have been detected in rodent cerebellum starting from postnatal day (PND) 4, meaning that the developing cerebellum is able to actively produce ROS. Moreover, administration of antioxidant/NOX inhibitor compounds, such as apocynin, has been demonstrated to decrease ROS levels in Purkinje cells [36]. However, so far, little is known about possible changes of NOX1 and NOX2 enzymes in this brain area following an early CNS insult leading to a later psychotic disease. Together with oxidative stress, increased inflammatory states and/or reduced anti-inflammatory pathways have been reported following ketamine administration [37,38,39]. Furthermore, the developing CNS has been described as being particularly vulnerable to enhanced peripheral and central inflammation following an external insult [40]. 

Together with its anti-inflammatory actions [41], celastrol, extracted from the medicinal plant *Tripterygium wilfordii*, has been described to have significant benefits in preventing neuropathological alterations observed in animal models of neurodegenerative diseases [42,43,44], through numerous mechanisms, including ROS level decrease [45]. In particular, celastrol has been characterized as an effective NOX enzyme inhibitor, with an increased potency against NOX1 and NOX2, acting via the suppression of the association between the enzymatic subunits, located in the cytosol, and the membrane flavocytochrome [46]. Importantly, no available reports investigate the effects of celastrol administration in animal models of psychosis. Moreover, no evidence has been previously published on the possible impact of celastrol administration during a crucial period of brain maturation, or on the development of a psychotic state following an early CNS insult.

A major challenge in the field of oxidative stress in the CNS is represented by the possibility to directly measure ROS production and release in this body district. Therefore, different indirect approaches have been used to quantify free radical amount in the CNS, including the analysis of 8-hydroxydeoxyguanosine (8-OHdG), a reliable marker of DNA oxidation levels [47,48].

Here, we investigated the impact of early celastrol administration on behavioral dysfunctions observed in adult mice exposed to subanesthetic doses of ketamine at PNDs 7, 9 and 11. The effects of this compound on ketamine-induced oxidative stress, as well as on NADPH oxidase expression alterations and PV levels in the cerebellum, were also assessed. Moreover, we also evaluated early celastrol effects on possible ketamine-induced changes of proinflammatory (Tumor Necrosis Factor alpha (TNF-α), interleukin-6 (IL-6) and interleukin-1 beta (IL-1β)), as well as anti-inflammatory [interleukin-10 (IL-10)] cytokines in the same brain region.

## 2. Results

### 2.1. Early Celastrol Administration Prevented Cognitive Dysfunctions in Adult Mice Exposed to Ketamine in Postnatal Life

**Figure 1:**
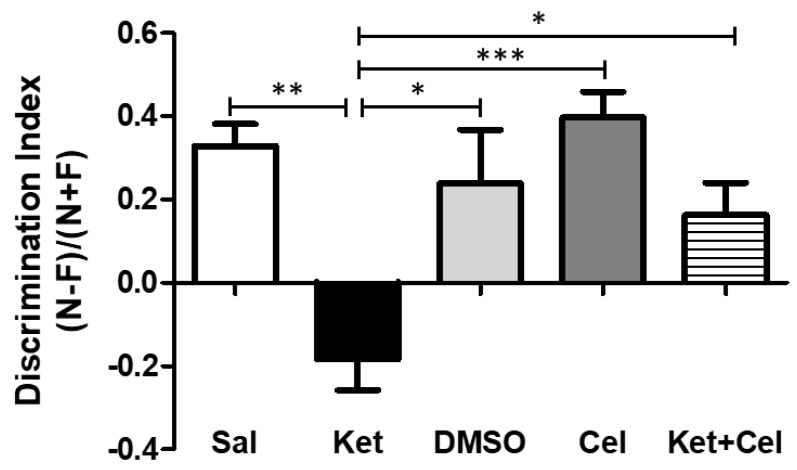
Celastrol administration in postnatal life prevented ketamine-induced cognitive dysfunctions, evaluated at adulthood. Discrimination index (N − F)/(N + F) (*N* = time spent in exploration of the novel object during the T2; F = time spent in exploration of the familiar object in the T2) in adult mice receiving saline (Sal, *n* = 6) or ketamine (Ket, *n* = 13) or a 50% DMSO in phosphate-buffered saline (PBS) (DMSO, *n* = 7) or celastrol (Cel, *n* = 6) or ketamine + celastrol (Ket + Cel, *n* = 14) at PNDs 7, 9 and 11. One Way ANOVA, followed by Tukey’s post hoc test F = 7.387, *** *p* < 0.001; ** *p* < 0.01; * *p* < 0.05.

To evaluate the possible effects of early celastrol administration on cognitive dysfunctions induced by ketamine exposure in postnatal life, we performed the Novel Object Recognition (NOR) test in 10 weeks mice. While no differences were detected in the discrimination index among saline, dimethyl sulfoxide (DMSO) and celastrol-treated mice, a significant decrease of this parameter was observed in adult mice who had received ketamine in postnatal life. Early celastrol administration to ketamine-treated mice was able to prevent this cognitive dysfunction (Figure 1, One Way Analysis of variance-ANOVA, followed by Tukey’s post hoc test F = 7.387, *p* < 0.01 ketamine vs. saline; *p* < 0.05 ketamine vs. DMSO and vs. ketamine + celastrol; *p* < 0.001 ketamine vs. celastrol; *p* > 0.05 saline vs. DMSO, celastrol and ketamine + celastrol; *p* > 0.05 DMSO vs. celastrol and ketamine + celastrol; *p* > 0.05 celastrol vs. ketamine + celastrol).

### 2.2. Early Celastrol Administration Prevented Locomotor Dysfunctions in Adult Mice Exposed to Ketamine in Postnatal Life

**Figure 2:**
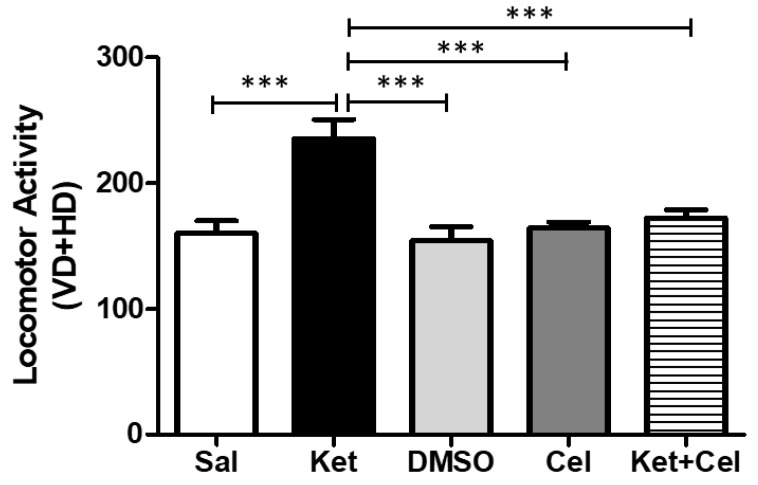
Celastrol administration in postnatal life prevented ketamine-induced increased in locomotor activity in later adulthood. Locomotor activity (VD = vertical displacements; HD = horizontal displacements) in adult mice receiving saline (Sal, *n* = 7) or ketamine (Ket, *n* = 13) or a 50% DMSO in PBS (DMSO, *n* = 7) or celastrol (Cel, *n* = 8) or ketamine + celastrol (Ket + Cel, *n* = 14) at PNDs 7, 9 and 11. One Way ANOVA, followed by Tukey’s post hoc test F = 10.34, *** *p* < 0.001.

To assess the possible impact of early celastrol administration on ketamine-induced locomotor alterations, we performed the Open Field (OF) test in adult mice. Ketamine administration in postnatal life significantly enhanced locomotor activity at 10 weeks of age, with respect to the saline, DMSO and celastrol-treated groups, within which no differences were observed. Celastrol co-administered with ketamine at PNDs 7, 9 and 11 was able to prevent the observed hyperlocomotion (Figure 2, One Way ANOVA, followed by Tukey’s post hoc test, F = 10.34, *p* < 0.001 ketamine vs. saline, DMSO, celastrol and ketamine + celastrol; *p* > 0.05 saline vs. DMSO, celastrol and ketamine + celastrol; *p* > 0.05 DMSO vs. celastrol and ketamine + celastrol; *p* > 0.05 celastrol vs. ketamine + celastrol). 

### 2.3. Early Celastrol Administration Prevented Social Behavior Dysfunctions in Adult Mice Exposed to Ketamine in Postnatal Life

**Figure 3:**
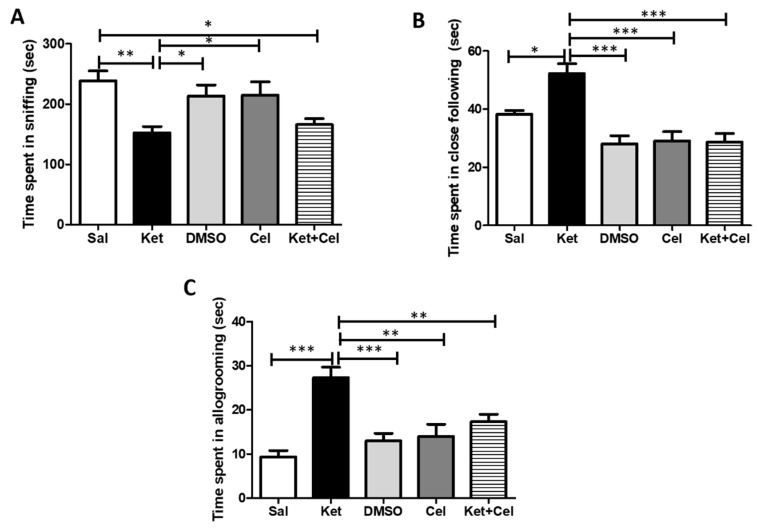
Celastrol administration in postnatal life prevented ketamine-induced social behavior dysfunctions in later adulthood. (**A**). Time spent in sniffing (seconds, sec) in adult mice receiving saline (Sal, *n* = 4) or ketamine (Ket, *n* = 8) or a 50% DMSO in PBS (DMSO, *n* = 4) or celastrol (Cel, *n* = 4) or ketamine + celastrol (Ket + Cel, *n* = 7) at PNDs 7, 9 and 11. One Way ANOVA, followed by Tukey’s post hoc test F = 6.856, ** *p* < 0.01; * *p* < 0.05. (**B**). Time spent in close following (seconds, sec) in adult mice receiving saline (Sal, *n* = 4) or ketamine (Ket, *n* = 7) or a 50% DMSO in PBS (DMSO, *n* = 4) or celastrol (Cel, *n* = 4) or ketamine + celastrol (Ket + Cel, *n* = 7) at PNDs 7, 9 and 11. One Way ANOVA, followed by Tukey’s post hoc test F = 13.10, *** *p* < 0.001; * *p* < 0.05. (**C**). Time spent in allogroming (seconds, sec) in adult mice receiving saline (Sal, *n* = 5) or ketamine (Ket, *n* = 6) or a 50% DMSO in PBS (DMSO, *n* = 4) or celastrol (Cel, *n* = 4) or ketamine + celastrol (Ket + Cel, *n* = 6) at PNDs 7, 9 and 11. One Way ANOVA, followed by Tukey’s post hoc test F = 12.50, *** *p* < 0.001; ** *p* < 0.01.

To investigate the effects of early celastrol administration on ketamine-induced social behavior impairments, we performed the Social Interaction (SI) test in adult mice. Animals receiving ketamine at PNDs 7, 9 and 11 showed a decrease in the sniffing time with respect to saline, DMSO- and celastrol-treated mice. A significant difference in this parameter was also observed in ketamine-treated mice who had also received celastrol in postnatal life compared to the saline group (Figure 3A, One Way ANOVA, followed by Tukey’s post hoc test, F = 6.856, *p* < 0.01 ketamine vs. saline; *p* < 0.05 ketamine vs. DMSO and celastrol; *p* < 0.05 ketamine + celastrol vs. saline; *p* > 0.05 saline vs. DMSO and celastrol; *p* > 0.05 DMSO vs. celastrol; *p* > 0.05 ketamine vs. ketamine + celastrol). Postnatal ketamine exposure caused a significant increase in the close following time, which was prevented by the concomitant treatment with celastrol (Figure 3B, One Way ANOVA, followed by Tukey’s post hoc test, F = 13.10, *p* < 0.05 ketamine vs. saline; *p* < 0.001 ketamine vs. DMSO, celastrol and ketamine + celastrol; *p* > 0.05 saline vs. DMSO, celastrol and ketamine + celastrol; *p* > 0.05 DMSO vs. celastrol and ketamine + celastrol; *p* > 0.05 celastrol vs. ketamine + celastrol). The same pattern was observed for the celastrol effects on ketamine-induced elevation of time spent in allogroming (Figure 3C, One Way ANOVA, followed by Tukey’s post hoc test, F = 12.50, *p* < 0.001 ketamine vs. saline and DMSO; *p* < 0.01 ketamine vs. celastrol and ketamine + celastrol; *p* > 0.05 saline vs. DMSO, celastrol and ketamine + celastrol; *p* > 0.05 DMSO vs. celastrol and ketamine + celastrol; *p* > 0.05 celastrol vs. ketamine + celastrol).

### 2.4. Early Celastrol Administration Prevented Oxidative Stress Increase in the Cerebellum of Adult Mice Exposed to Ketamine in Postnatal Life

**Figure 4:**
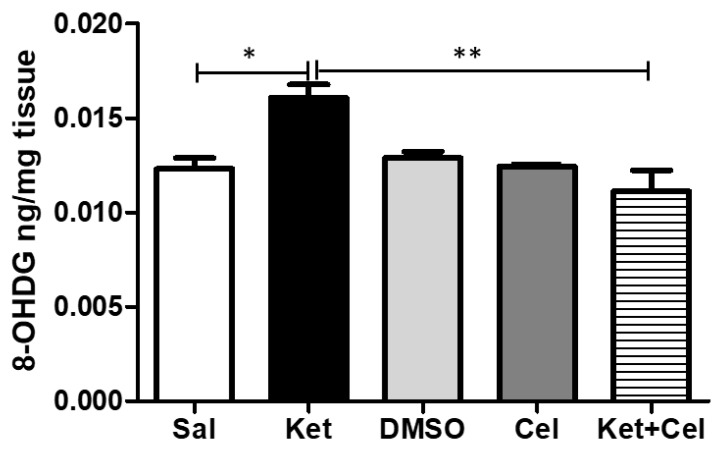
Celastrol administration in postnatal life prevented ketamine-induced oxidative stress in the cerebellum in later adulthood. 8-OHdG levels (ng/mg tissue) in the cerebellum of adult mice receiving saline (Sal, *n* = 3) or ketamine (Ket, *n* = 5) or a 50% DMSO in PBS (DMSO, *n* = 3) or celastrol (Cel, *n* = 3) or ketamine + celastrol (Ket + Cel, *n* = 5) at PNDs 7, 9 and 11. One Way ANOVA, followed by Tukey’s post hoc test F = 6.361 * *p* < 0.05; ** *p* < 0.01.

To assess the effects of early celastrol administration on ketamine-induced oxidative stress in the cerebellum of adult mice, we quantified 8-OHdG levels in this brain region. Mice receiving ketamine at PNDs 7, 9 and 11 showed a significant elevation of this biomarker of oxidative stress with respect to saline-treated animals whose 8-OHdG amount was comparable to the one of the DMSO and celastrol-treated animals. Early celastrol administration was able to prevent ketamine-induced enhancement of this biomarker (Figure 4, One Way ANOVA, followed by Tukey’s post hoc test, F = 6.361, *p* < 0.05 ketamine vs. saline; *p* < 0.01 ketamine vs. ketamine + celastrol; *p* > 0.05 saline vs. DMSO, celastrol and ketamine + celastrol; *p* > 0.05 DMSO vs. celastrol and ketamine + celastrol; *p* > 0.05 celastrol vs. ketamine + celastrol).

### 2.5. Early Celastrol Administration Decreased NOX1 Levels in the Cerebellum of Adult Mice Per Se and Following Ketamine Exposure

**Figure 5:**
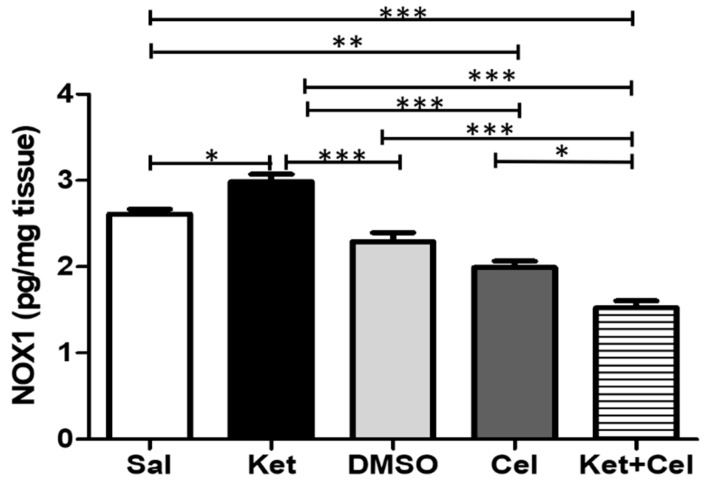
Celastrol administration in postnatal life decreased NOX1 levels in the cerebellum of adult mice. NOX1 levels (pg/mg tissue) in the cerebellum of adult mice receiving saline (Sal, *n* = 3) or ketamine (Ket, *n* = 5) or a 50% DMSO in PBS (DMSO, *n* = 3) or celastrol (Cel, *n* = 3) or ketamine + celastrol (Ket + Cel, *n* = 5) at PNDs 7, 9 and 11. One Way ANOVA, followed by Tukey’s post hoc test F = 50.30, * *p* < 0.05; ** *p* < 0.01; *** *p* < 0.001.

To evaluate the effects of early celastrol administration on ketamine-induced NADPH oxidase alterations in the cerebellum, we measured NOX1 and NOX2 levels in this area. NOX1 amount was significantly increased by ketamine administration in postnatal life. Celastrol, injected as single treatment at the same time point, reduced NOX1 levels compared to both saline or ketamine-treated mice. The amount of this NADPH oxidase isoform was further reduced when celastrol was administered early to ketamine-treated animals (Figure 5, One Way ANOVA, followed by Tukey’s post hoc test F = 50.30, *p* < 0.05 ketamine vs. saline and ketamine + celastrol vs. celastrol; *p* < 0.01 celastrol vs. saline; *p* < 0.001 ketamine + celastrol vs. saline and vs. DMSO and ketamine vs. DMSO, celastrol and ketamine + celastrol; *p* > 0.05 saline vs. DMSO).

**Figure 6:**
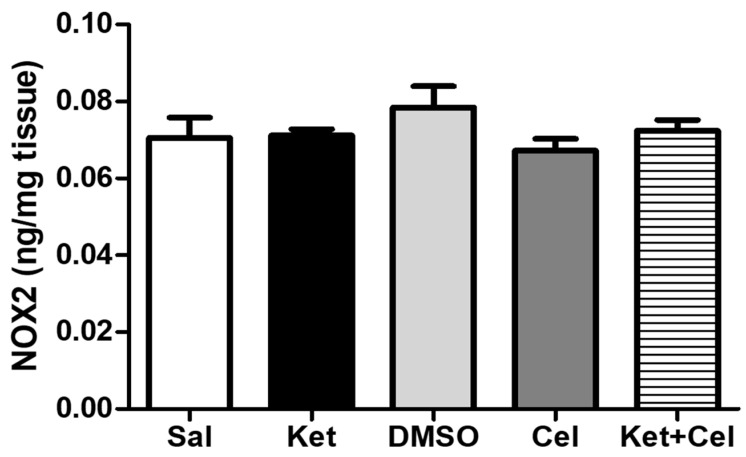
NOX2 levels were not altered in the cerebellum of adult mice exposed to ketamine in postnatal life. NOX2 levels (ng/mg tissue) in the cerebellum of adult mice receiving saline (Sal, *n* = 3) or ketamine (Ket, *n* = 5) or a 50% DMSO in PBS (DMSO, *n* = 3) or celastrol (Cel, *n* = 3) or ketamine + celastrol (Ket + Cel, *n* = 5) at PNDs 7, 9 and 11. One Way ANOVA, followed by Tukey’s post hoc test F = 1.158 *p* > 0.05 for all comparisons.

Ketamine administration at PNDs 7, 9 and 11 did not significantly alter NOX2 amount in the cerebellum of adult mice, and no differences in the levels of this NADPH oxidase isoform were detected among all the other experimental groups (Figure 6, One Way ANOVA, followed by Tukey’s post hoc test F = 1.158, *p* > 0.05 for all comparisons). 

**Figure 7:**
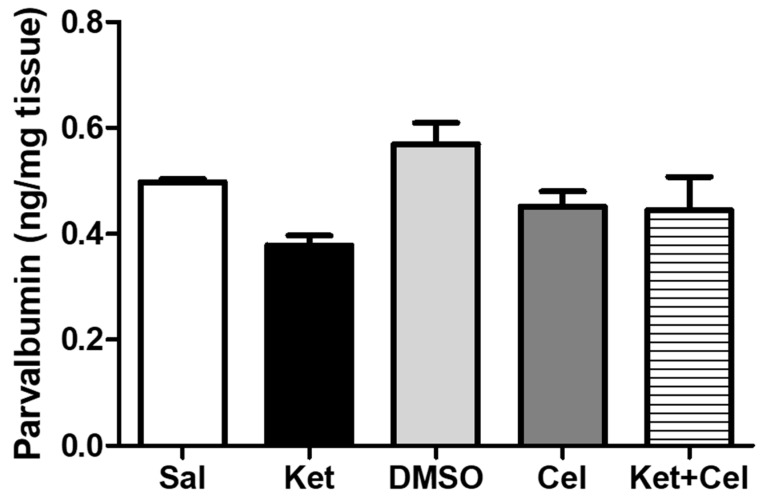
PV levels were not altered in the cerebellum of adult mice exposed to ketamine in postnatal life. PV levels (ng/mg tissue) in the cerebellum of adult mice receiving saline (Sal, *n* = 3) or ketamine (Ket, *n* = 5) or a 50% DMSO in PBS (DMSO, *n* = 3) or celastrol (Cel, *n* = 3) or ketamine + celastrol (Ket + Cel, *n* = 5) at PNDs 7, 9 and 11. One Way ANOVA, followed by Tukey’s post hoc test F = 2.632, *p* > 0.05 for all comparisons.

The same was observed for cerebellar PV levels (Figure 7, One Way ANOVA, followed by Tukey’s post hoc test, F = 2.632, *p* > 0.05 for all comparisons).

### 2.6. Early Celastrol Administration Did not Prevent TNF-α Increase in the Cerebellum of Adult Mice Exposed to Ketamine in Postnatal Life

**Figure 8:**
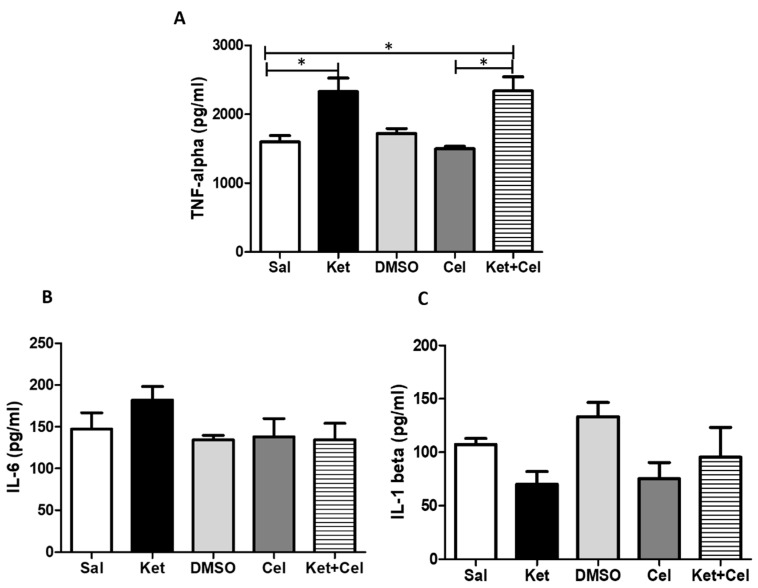
Celastrol administration in postnatal life did not prevent ketamine-induced TNF-α increase in the cerebellum in later adulthood. (**A**). TNF-α levels (pg/mg tissue) in the cerebellum of adult mice receiving saline (Sal, *n* = 3) or ketamine (Ket, *n* = 5) or a 50% DMSO in PBS (DMSO, *n* = 3) or celastrol (Cel, *n* = 3) or ketamine + celastrol (Ket + Cel, *n* = 5) at PNDs 7, 9 and 11. One Way ANOVA, followed by Tukey’s post hoc test F = 7.382, * *p* < 0.05. (**B**). IL-6 levels (pg/mg tissue) in the cerebellum of adult mice receiving saline (Sal, *n* = 3) or ketamine (Ket, *n* = 5) or a 50% DMSO in PBS (DMSO, *n* = 3) or celastrol (Cel, *n* = 3) or ketamine + celastrol (Ket + Cel, *n* = 5) at PNDs 7, 9 and 11. One Way ANOVA, followed by Tukey’s post hoc test F = 1.444, *p* > 0.05. (**C**). IL-1β levels (pg/mg tissue) in the cerebellum of adult mice receiving saline (Sal, *n* = 3) or ketamine (Ket, *n* = 5) or a 50% DMSO in PBS (DMSO, *n* = 3) or celastrol (Cel, *n* = 3) or ketamine + celastrol (Ket + Cel, *n* = 5) at PNDs 7, 9 and 11. One Way ANOVA, followed by Tukey’s post hoc test F = 2.103 *p* > 0.05.

To investigate the effects of early celastrol administration on ketamine-induced inflammation in the cerebellum, we measured levels of TNF-α, IL-6 and IL-1β in this brain area. Ketamine administration in postnatal life determined an enhancement of cerebellar TNF-α in later adulthood compared to controls which showed comparable TNF-α amount with respect to the DMSO and celastrol-treated groups. Increased TNF-α were also detectable in adult mice receiving both ketamine and celastrol at PNDs 7, 9 and 11 (Figure 8A, One Way ANOVA, followed by Tukey’s post hoc test F = 7.382, *p* < 0.05 ketamine vs. saline, saline vs. ketamine + celastrol and celastrol vs. ketamine + celastrol), whereas no significant alterations in the amount of IL-6 (Figure 8B, One Way ANOVA, followed by Tukey’s post hoc test F = 1.444 *p* > 0.05 for all comparisons) and IL-1β (Figure 8C, One Way ANOVA, followed by Tukey’s post hoc test F = 2.103 *p* > 0.05 for all comparisons) in the same brain region were found.

### 2.7. Early Celastrol Administration Prevented IL-10 Decrease in the Cerebellum of Adult Mice Exposed to Ketamine in Postnatal Life

**Figure 9:**
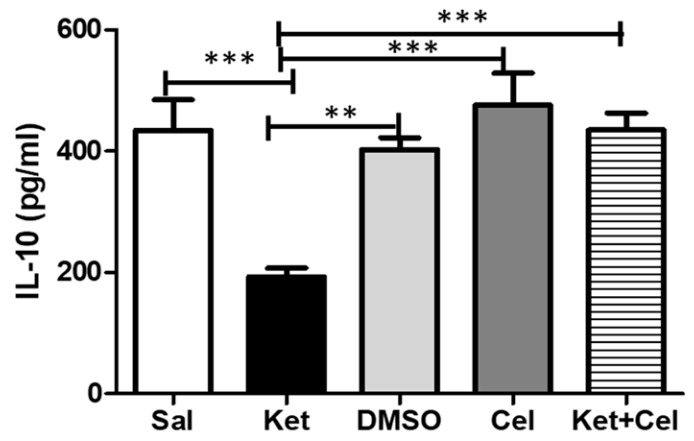
Celastrol administration in postnatal life prevented ketamine-induced IL-10 decrease in the cerebellum in later adulthood. IL-10 levels (pg/mL) in the cerebellum of adult mice receiving saline (Sal, *n* = 3) or ketamine (Ket, *n* = 5) or a 50% DMSO in PBS (DMSO, *n* = 3) or celastrol (Cel, *n* = 3) or ketamine + celastrol (Ket + Cel, *n* = 5) at PNDs 7, 9 and 11. One Way ANOVA, followed by Tukey’s post hoc test F = 15.19, *** *p* < 0.001, ** *p* < 0.01.

To assess the effects of early celastrol administration on ketamine-induced decrease of anti-inflammatory cytokines in the cerebellum, we quantified IL-10 levels in this brain region. Mice administered with ketamine at PNDs 7, 9 and 11 showed reduced IL-10 amounts in later adulthood compared to saline-treated animals, whose levels of this cytokine were comparable to the ones detected in mice receiving DMSO or celastrol. Early celastrol administration in ketamine-treated animals was able to prevent IL-10 reduction in the cerebellum (Figure 9, One Way ANOVA, followed by Tukey’s post hoc test F = 15.19, *p* < 0.001 ketamine vs. saline, ketamine vs. celastrol and ketamine vs. ketamine + celastrol; *p* < 0.01 ketamine vs. DMSO).

## 3. Discussion

In this work, we demonstrated that early celastrol administration prevented discrimination ability dysfunctions, locomotor activity alterations and social behavior impairment in adult mice that had received ketamine at PNDs 7, 9 and 11. Previously published in vivo studies investigating possible beneficial effects of celastrol on CNS disorders have mainly regarded neurodegenerative disorders, including Alzheimer’s disease [43,44,45], Parkinson’s diseases [49,50,51], amyotrophic lateral sclerosis [42,52] and multiple sclerosis [53,54], epilepsy [55,56], cerebral ischemia and ischemic stroke [57,58,59] as well as traumatic brain injury [60,61]. One in vitro report indirectly investigated the impact of celastrol on the expression of specific genes, such as Fragile X Mental Retardation 1 (FMR1), linked to different psychiatric diseases, including schizophrenia [62]. Therefore, a novelty of our study with respect to the existing literature in the field is related to the evaluation of the effects of celastrol in psychotic disease by using a mouse model of the disorder. Importantly, this was obtained by negatively impacting the process of brain maturation with an early detrimental insult, represented by ketamine administration. Indeed, it has been reported that the developing brain is more vulnerable to the neurotoxicity induced by this psychoactive compound compared to the mature brain, in terms of enhanced neuronal cell death, neurogenesis alterations, disruptions of γ-aminobutyric acid (GABA)ergic interneuron development, altered NMDA-R expression, impaired synaptogenesis and increased oxidative stress production [63]. These disturbances during a critical period of brain maturation, i.e., the first 2–3 weeks of life in rodents, when brain growth spurt occurs, have been reported to trigger brain dysfunctions later in life, resulting finally in psychotic-like neuropathological and behavioral alterations [64]. Thus, our observations suggest that early administration of celastrol concomitantly to a brain insult might block the detrimental effects of ketamine with respect to the development of CNS and stop the progression of cerebral damage.

Decreased discrimination ability in rodents has been considered a behavioral feature mimicking the cognitive dysfunctions observed in psychotic patients [6]. Our findings regarding the preventive effects of early celastrol administration on ketamine-induced decrease in cognitive functions are in line with previous observations reporting a beneficial impact of this compound on learning and memory dysfunctions induced by metabolic alterations [65] or by neurodegenerative processes, induced by aggregation of specific proteins [45,66]. However, these studies were mainly focused on the behavioral effects of celastrol administration in adult life or even later, when the CNS insults leading to brain damage might have already been consolidated.

Elevations in locomotor activity in rodents are known to mimic the psychomotor agitation observed in subjects suffering from psychosis [5]. In our experimental conditions, early celastrol-treated mice, also exposed to ketamine in postnatal life, did not show an increase in locomotor activity with respect to the other experimental groups. Accordingly, the beneficial effects of celastrol on locomotor activity dysfunctions have been previously described in animal models of epilepsy, where motor function alterations were rapidly reduced by celastrol administration [67].

In this work, we also showed that ketamine administration at PNDs 7,9 and 11 induced dysfunctions in social behavior at adulthood. In particular, we reported decreased sniffing time in ketamine-treated mice with respect to controls. Together with its relation to social hierarchy in rodents, the sniffing behavior has been shown to be related to the establishment of normal social interactions [68]. Importantly, decreased social interactions in rodents have been paralleled to a negative symptom observed in psychotic patients, i.e., the social withdrawal [5]. Our findings are in line with previous work reporting a decreased sniffing time in rats treated with another NMDA-R antagonist, phencyclidine [69], together with a positive effects of antipsychotic treatment in reverting this social deficit [70]. Moreover, decreased duration of sniffing was observed in animal models of neuropsychiatric diseases also characterized by psychotic symptoms, such as autism [71]. In our experimental conditions, mice receiving ketamine in postnatal life also showed increased time spent in close-following and allogrooming. Close following is generally considered a mutual investigation behavior, while allogroming has been described as a standard behavior of altruism and reciprocal cooperation [72]. However, despite their general classification as non-aggressive behaviors, elevations in these social outcomes have been associated with subordination of the partner and abnormal dominance establishment [73], which could be seen as aggressive-like behaviors [74]. Accordingly, Becker et al. described a decrease in non-aggressive behavior in ketamine-treated rats [72]. Our findings might appear to be in contradiction with a previous work reporting that ketamine ameliorates aggressive-like behavior induced by neonatal maternal separation in mice [75]. However, in this study, lower doses of ketamine (15 mg/kg) were used and the administration time (post-natal days 35–49) was not comparable to those followed in our research procedure. In our experimental conditions, celastrol did not show beneficial effects on the ketamine-induced social withdrawal at adulthood but was able to prevent the observed increase in aggressive-like behavior. This is in line with previous findings reporting beneficial effects of antioxidant therapies in attenuating aggressive behavior induced by different stimuli [76] and describing aggressivity enhancement in mice with a genetic reduction of antioxidant functions [77]. In apparent contrast with our findings, Phensy and co-workers demonstrated that antioxidant treatment with N-acetyl cysteine was able to prevent social interaction dysfunctions induced by ketamine administration during postnatal life. However, in this work, administration of this antioxidant compound was performed throughout the entire period of brain development. Thus, we cannot exclude that prolonged administration of celastrol during brain maturation might also have an impact on social withdrawal observed at adulthood. 

In our study, we found that early celastrol administration prevented elevations in cerebellar oxidative stress observed in mice treated with ketamine in postnatal life. The cerebellum has been gaining increasing importance in the pathogenic mechanisms underlying the development of psychosis [11,78,79,80] and of other psychiatric diseases, clinically characterized by psychotic symptoms [81]. In addition to the ketamine-induced detrimental effects on the prefrontal cortex [22,82,83], the negative impact of this NMDA-R antagonist also on the developing cerebellum has been shown in non-human primates [84]. In good agreement with our observations, previous works have reported increased direct and indirect biomarkers of oxidative stress in the cerebellum of animal models of neuropsychiatric disorders [85,86]. In particular, Filiou and co-workers described cerebellar oxidative stress-induced structural alterations in the G72/G30 transgenic schizophrenia mouse model [87]. Moreover, antipsychotic medication has been demonstrated to inhibit the activity of specific enzymes, which can also produce free radicals in rodent cerebellum [88,89]. The increased oxidative stress observed in this brain region may also be considered a possible trigger of the cerebello-thalamo-cortical network dysfunctions which have been described as predictors of disease progression in individuals at ultra-high risk for psychosis [12,90]. In support of this concept, interesting lines of evidence describe a positive effects of antioxidant treatments in preventing cerebellar dysfunctions observed in neuropsychiatric diseases also characterized by psychotic symptoms, such as autism spectrum disorder [91]. 

An important finding of our study consists in the observed increased cerebellar NOX1 levels in adult mice who had received ketamine in postnatal life, whereas NOX2 amount was not affected by this early detrimental insult. A physiological role of the NADPH oxidase enzymatic family in different stages of cerebellum development has been previously described [36]. Moreover, Olguín-Albuerne and Morán reported a key role of NADPH oxidase-derived ROS in controlling the development of cerebellar granule neurons during brain maturation [92]. However, in vitro and in vivo evidence highlighted a crucial role of NOX enzymes in the development of structural and functional alterations in cerebellum following different insults [93,94,95]. Increased NOX1 enzyme expression and activity has been implicated not only in the pathogenesis of neurodegenerative disorders [96,97,98], but also in neurotoxic processes mediated by sustained microglia activation [99]. Thus, the observed NOX1 increase following postnatal ketamine administration should also be considered in the context of the effects that this NMDA-R antagonist has on the inflammatory states of the brain. Supporting this perspective, it has been reported that exposure to subanesthetic doses of ketamine is able to activate neuroinflammatory pathways [83] and to induce microglia activation in rodent brains [100]. In our experimental conditions, early celastrol administration was able per se to decrease NOX1 levels in the cerebellum of adult mice which did not receive ketamine in postnatal life. Although speculative, a possible explanation for this result could be related to possible celastrol effects on other ketamine-independent events occurring in mature brain and implicating a role of the NADPH oxidase system, such as protein aggregation [101] or specific heat shock proteins expression and/or activation [102,103]. Hence, in the presence of a neurodetrimental insult, i.e., ketamine, early celastrol administration was able to further lower cerebellar NOX1 levels. With respect to these findings, additional investigations are needed to further unravel molecular mechanisms of actions of celastrol and its possible impact on NOX1 enzyme expression. Indeed, in this context, a limitation of this study is represented by the absence of the evaluation of the enzyme activation in the cerebellum. The lack of NOX2 increase following postnatal ketamine exposure observed in our experimental conditions could be explained by a region-specific effect of this NMDA-receptor antagonist in inducing an enhancement of this NADPH oxidase isoform. In line with this hypothesis, Zhang and co-workers previously described that cortical NOX2 was upregulated in adult rats treated with ketamine from PND6 to PND8 [20]. Moreover, in further support, an interesting study of Boczek et al. analyzed the effects of repeated ketamine administration on different brain areas, i.e., cortex, cerebellum, hippocampus and striatum, revealing region-specific effects of this NMDA-R antagonist [104]. However, we cannot totally exclude that the observed celastrol effects might be related to other pathways, other than the inhibition of NOX enzymes, finally resulting in decreased ROS levels, such as the enhancement of antioxidant capacity [105], the increase of antioxidant enzyme activity [106] and the targeting of mitochondria respiratory chain [107].

Decreased PV levels and loss of phenotype of PV-positive interneurons have been described in brain regions other than cerebellum, such as prefrontal cortex and hippocampus, in pharmacologic and non-pharmacologic animal models of psychosis [22,108]. With respect to this issue, a novelty of the present study is the absence, at least in our experimental conditions, of the reduction of PV amount in the cerebellum of adult mice administered with ketamine in the early stages of life, suggesting a region-specific effect of this NMDA-R antagonist. Moreover, our findings should also be considered in the light of the link existing between NADPH oxidases and PV. Indeed, NOX2 enzyme alterations have been reported to mediate cortical PV changes induced by different neurodetrimental insults, such as ketamine administration [34] or traumatic brain injury [109]. Thus, the lack of PV alterations observed in our experimental conditions might be related to the absence of NOX2 changes in the same brain region, suggesting a different mechanism of action underlying ketamine effects in the cerebellum with respect to what observed in the prefrontal cortex. 

Here, we also showed that ketamine administration in early life stages caused increased levels of a specific proinflammatory cytokine, the TNF-α, in the cerebellum, without affecting cerebellar levels of IL-6 and IL-1β. Behavioral manifestations in psychiatric disorders such as schizophrenia and autism have been reported to be sustained by early neuroinflammatory processes which involve specific brain regions, including the cerebellum [110]. Moreover, patients with first psychotic episode, drug-naive schizophrenia, and subjects at ultra-high risk of psychosis have been described to share altered cerebellar-default mode network connectivity which appears to be modulate by inflammation in this brain region [111]. Moreover, in good agreement with our findings, previous evidence has reported a crucial role of TNF-α in regulating ketamine-induced neurotoxicity in the hippocampus [112,113], which is known to be functionally connected with the cerebellum [114,115]. In our experimental conditions, early celastrol administration was not able to prevent the ketamine-induced TNF-α increase in the cerebellum. This finding might appear in apparent contradiction with previous lines of evidence showing an effect of this compound in lowering TNF-α in monocytes and macrophages [45], as well as in the brain of animal models of neurodegenerative disorders, such as Alzheimer’s disease [116,117], amyotrophic lateral sclerosis [42] and Parkinson’s disease [50]. However, in most of the animal models on which celastrol has previously been tested for the evaluation of its effects on TNF-α, the pathological and/or neurotoxic insult leading to the neurodegenerative condition mainly occurred at adulthood. Moreover, other routes of administration (such as the oral one), as well as different doses and considered brain regions might also explain our findings. Further research is certainly needed to highlight possible different effects of celastrol on pro-inflammatory cytokines based on the time of the insult occurring in the brain. The lack of ketamine-induced cerebellar IL-6 increase that we observed might also be considered in the light of the unaltered NOX2 and PV expression we found in the same brain region. Indeed, a molecular association between IL-6, NOX2 and PV has been previously reported in the ketamine model of psychosis [35]. 

In this study, we also reported that early celastrol administration prevented ketamine-induced decrease of IL-10, an anti-inflammatory cytokine, in the cerebellum. Accordingly, an imbalance between pro-inflammatory and anti-inflammatory cytokines has been described in both schizophrenia [118] and other psychiatric disorders characterized by psychotic symptoms, such as bipolar disorders [119]. Intriguingly, IL-10 has been described as the most important player both in the resolution of the inflammatory cascade [120] and in the protection against possible detrimental effects following a neurotoxic insults [121,122]. Moreover, a key role of this anti-inflammatory cytokine in preventing glutamate-mediated cerebellar granule cell death has been reported [123], together with the regulation of synapses formation and functioning in the developing brain [124]. Thus, we could hypothesize that, at least in our experimental conditions, early celastrol administration might exert a protective effect against a neurotoxic insult, represented by ketamine, on the developing cerebellum, acting also on the anti-inflammatory pathways related to IL-10.

In conclusion, our study suggests that early NOX inhibition by celastrol during a crucial period of CNS maturation can prevent the development of psychotic-like behavioral dysfunctions, the increased oxidative stress and the IL-10 reduction in the cerebellum of adult mice exposed to an early neurodetrimental insult, i.e., ketamine. This might open new pharmacological insights into the possible use of this compound for neuroprotective purposes during brain development. 

## 4. Materials and Methods

### 4.1. Animals

Mice were housed at constant room temperature (22 ± 1 °C) and relative humidity (55 ± 5%) under a 12 h light/dark cycle (lights on from 7:00 AM to 7:00 PM), with free access to food and water. Experimental procedures involving animals and their care were performed in conformity with the institutional guidelines of the Italian Ministry of Health (D.Lgs. n.26/2014), the Guide for the Care and Use of Laboratory Animals: Eight Edition, the Guide for the Care and Use of Mammals in Neuroscience and Behavioral Research (National Research Council, 2004), the Directive 2010/63/EU of the European Parliament and of the Council of 22 September 2010 on the protection of animals used for scientific purposes, as well as the ARRIVE guidelines. We daily monitored animal welfare during the entire period of experimental procedures. All efforts were made to minimize the number of animals used and their suffering. The experimental protocol was approved by the Italian Ministry of Health (approval number 679/2017-PR, protocol n. B2EF8.17).

### 4.2. Experimental Design

Five C57/Bl6 adult male mice and 10 adult females (Envigo, San Pietro al Natisone, Italy) weighing 25–30 g (8–10 weeks of age) were mated (one male and two females per cage). 

Male pups were divided into the following five experimental groups:

1. pups administered with saline (10 mL/kg i.p.);
2. pups administered with ketamine (Sigma-Aldrich Corporation, Saint Louis, MO, US; 30 mg/kg i.p., dissolved in saline) [10,33];
3. pups administered with celastrol (Sigma Aldrich, Milano, Italy; 1 mg/kg i.p., dissolved in 50% DMSO/PBS) [43];
4. pups administered with a 50% DMSO/PBS solution (5 mL/kg i.p.)—we have referred to this treatment throughout the text as “DMSO”;
5. pups administered with ketamine (30 mg/kg i.p., dissolved in saline, injected in the right side of the peritoneum) and celastrol (1 mg/kg i.p., dissolved in 50% DMSO/PBS, injected in the left side of the peritoneum)—we have referred to this treatment throughout the text as “ketamine + celastrol”.

The above-mentioned treatments were repeated at PNDs 7, 9 and 11. 

All pups were grown until adulthood, i.e., 10 weeks of age, when behavioral tests were performed. Immediately after, mice were euthanized by cervical dislocation for the collection of cerebella on which neurochemical and biomolecular analysis were conducted. The tissue was frozen in isopentane and stored at −80 °C until analysis was performed.

Body weight gain during the experimental protocol was calculated as the difference between body weight at PND 7 (the time of the first ketamine and/or celastrol injection) and body weight at 10 weeks of age (the time at which the behavioral tests were performed). No statistical differences were detected among the experimental groups (Supplementary Material A). Moreover, body weight at the time of the behavioral tests (10 weeks of age) was comparable among experimental groups (Supplementary Material B). No evident signs of hair loss and/or alopecia were observed during the experimental protocol for all the animals included in this study. 

### 4.3. Behavioral Tests

#### 4.3.1. NOR Test

The NOR test was performed as previously described [19,125] in a squared plastic-made arena (40 cm × 40 cm × 40 cm). For the habituation, mice were allowed to freely explore the arena for 10 min over five days. Mice were acclimatized to the testing room for one hour prior the beginning of the test. The test included two trials (training trial, T1 and testing trial, T2) of 3 min with an intertrial time of 1 min [126,127]. In T1, mice were put in the center of the arena and left free to explore two identical objects (two white light bulbs, fixed on the floor of the arena by velcro) for 3 min. In the testing trial (T2), one of the light bulbs was substituted with a novel object (a light blue plastic-made brick). At the beginning of the experimental procedure and between T1 and T2, the objects were cleaned with 20% *v*/*v* ethanol to remove any olfactory cues. Moreover, the arena was cleaned each time to remove mouse feces. Both T1 and T2 were videorecorded using a fixed camera. Then, an investigator, blind to the identity of the tested mouse, analyzed the animal behavior, including in the scoring of object sniffing and touching, as well as having moved the vibrissae while directing the nose toward the object at a distance of 1 cm. The following behaviors were not considered: sitting on, leaning against, and chewing the objects. The discrimination index was calculated using the following formula: (N − F)/(N + F) (*N* = times spent in exploration of the novel object during the T2; F = times spent in exploration of the familiar object in the T2) [19].

#### 4.3.2. OF Test

The OF test was performed as previously described [128], in a square plastic arena (40 cm × 40 cm × 40 cm), virtually divided into nine equal squares with black horizontal and vertical lines [129]. Mice were acclimatized to the testing room for one hour prior the beginning of the test. For the habituation, mice were allowed to freely move into the arena for 10 min over five days. The day of the test, mice were initially placed in the same corner and then left to move freely in the arena for 5 min. The experimental procedures were videorecorded using a fixed camera and then analyzed by a blind investigator who manually scored as spontaneous locomotor activity the total of horizontal and vertical displacements performed during the test (squares crossed with the four paws).

#### 4.3.3. SI Test

The SI test was performed, as previously described [130,131,132], in a plexiglass rectangular cage (45cm × 30cm × 25cm), located under a fixed camera. Briefly, 24 h before, as well as on the morning of the test, the cages were cleaned, the testing mouse was weighed in order to choose an appropriate intruder, which was labelled with a white, sticking tape on the tail. Mice were acclimatized to the testing room for one hour prior the beginning of the test. The testing mouse was left undisturbed in the cage for 15 min. Then, the intruder was introduced, and the social behavior was videorecorded for 10 min. Analysis of behavior was conducted by a blind researcher and the following parameters were considered for the scoring: time (seconds) spent by the testing mouse in sniffing the intruder, time (seconds) spent by the testing mouse in close following the intruder and time (seconds) spent by the testing mouse in the allogrooming to the intruder. 

### 4.4. Enzyme-Linked Immunosorbent Assays (ELISAs)

Samples were homogenized in 10 volumes of PBS with protease inhibitors, as previously described [133,134]. Commercially available ELISA kits were used for measurement of 8-OHdG (JaICA, Shizuoka, Japan), NOX2 (MyBiosource, San Diego, CA, USA), NOX1 (MyBiosource, San Diego, CA, USA), PV (MyBiosource, San Diego, CA, USA), TNF-α (MyBiosource, San Diego, CA, USA), IL-6 (MyBiosource, San Diego, CA, USA), IL-1β (MyBiosource, San Diego, CA, USA) and IL-10 (MyBiosource, San Diego, CA, USA) in the cerebellum, according to the manufacturer’s instructions. Each sample analysis was performed in duplicate to avoid intra-assay variations.

### 4.5. Blindness of the Study

Researchers performing data analysis were blind with respect to the treatment conditions. The blindness was maintained until the end of the analysis process.

### 4.6. Statistical Analysis

GraphPad 5.0 software for Windows was used to perform statistical analyses. Data were analyzed by One Way ANOVA, followed by Tukey’s post hoc test. For all tests, a *p* value < 0.05 was considered statistically significant. Results are expressed as means ± mean standard error (SEM).

## Supplementary Materials

The Supplementary Materials are available online at https://www.mdpi.com/1420-3049/24/21/3993/s1.
