# Supplementary material for: Early Celastrol Administration Prevents Ketamine-Induced Psychotic-Like Behavioral Dysfunctions, Oxidative Stress and IL-10 Reduction in The Cerebellum of Adult Mice
Source: Molecules. 2019 Nov 5;24(21):3993. doi: 10.3390/molecules24213993 (PMC6864687; doi:10.3390/molecules24213993)
Supplement: Supplementary file 1 [file molecules-24-03993-s001.pdf]

A) Body weight gain (g)

Saline (n=7): 21.97 ± 0.3264

Ketamine (n=13): 21.95 ± 0.3267

DMSO (n=7): 22.67 ± 0.4052

Celastrol (n=8): 22.18 ± 0.4495

Ketamine+Celastrol (n=14): 22.69 ± 0.3525

One Way ANOVA, followed by Tukey's post hoc test $F = 0.9866$, $p > 0.05$ for all comparisons

B) Body weight (g) at the time of behavioral tests (10 weeks of age)

Saline (n=7): 26.83 ± 0.3092

Ketamine (n=13): 26.65 ± 0.2480

DMSO (n=7): 27.17 ± 0.3914

Celastrol (n=8): 26.95 ± 0.4840

Ketamine+Celastrol (n=14): 27.34 ± 0.2401

One Way ANOVA, followed by Tukey's post hoc test $F = 0.8986$, $p > 0.05$ for all comparisons
